# Blood-Borne Hepatitis in Opiate Users in Iran: A Poor Outlook and Urgent Need to Change Nationwide Screening Policy

**DOI:** 10.1371/journal.pone.0082230

**Published:** 2013-12-02

**Authors:** Behnam Honarvar, Neda Odoomi, Mohsen Moghadami, Parvin Afsar Kazerooni, Alireza Hassanabadi, Parvin Zare Dolatabadi, Ehsan Farzanfar, Kamran Bagheri Lankarani

**Affiliations:** 1 Health Policy Research Center, Shiraz University of Medical Sciences, Shiraz, Iran; 2 Health Department, Shiraz University of Medical Sciences, Shiraz, Iran; 3 HIV/AIDS Research Center, Shiraz University of Medical Sciences, Shiraz, Iran; 4 Behavioral Counseling Center, HIV/AIDS Research Center, Shiraz University of Medical Sciences, Shiraz, Iran; 5 Behavioral Counseling Center, Shiraz University of Medical Sciences, Shiraz, Iran; 6 Diagnostic Laboratory Sciences and Technology Research Center, School of Paramedical Sciences, Shiraz University of Medical Sciences, Shiraz, Iran; University of Cincinnati College of Medicine, United States of America

## Abstract

**Objective:**

Iran has the highest rate of opiate use worldwide. However, most opiate users are not screened for hepatitis virus infections. This study aimed to provide accurate, detailed data on the size of the opiate user population at risk of developing these infections.

**Method:**

This seroprevalence study was conducted in the city of Shiraz, southern Iran. All participants were screened for HBV, HCV and HIV infection. The data were analyzed with SPSS.

**Result:**

Among 569 participants, 233 (40.9%) were injection drug users (IDU), 369 (64.8%) were heterosexual, 84 (14.7%) were bisexual and 15 (2.6%) were homosexual. One hundred nine (19.1%) were HCV antibody-positive, 18 (3.1%) were HBS antigen-positive, 72 (12.6%) were HBc antibody-positive and 23 (4%) were HIV-positive. Among IDU compared to non-IDU, positivity rates for HBS antigen (5.5 vs 1.4%), HBc antibody (22.7 vs 5.6%), HCV antibody (40.3 vs 4.4%) and HIV (7.7 vs 1.4%) were higher (*P* < 0.05). Most patients with HBV (80.7%) and HCV infection (83.4%) were HIV-negative. In the cumulative analysis, only history of imprisonment was a statistically significant determinant of infection by HCV or HBV in opiate users.

**Conclusion:**

The current policy of screening only HIV-positive drug users for HBV and HCV in Iran misses most cases of HBV and HCV infection. We therefore recommend urgent revision of the nationwide protocol by the Ministry of Health in Iran to implement routine screening of all opiate users and especially IDU for these viruses, regardless of their HIV status.

## Introduction

Iran has one of the highest rates of opiate use in the world, and the use of opium and heroin increased in 2010 compared to earlier figures [1]. Among the population of Iran between the ages of 15 and 64 years, 2.7% (1.1% to 5.9%) are estimated to use opiates [2]; 40% of these users consume opium, and the rest mainly consume heroin [3]. The main and most worrisome impact of opiate use is on health. Long-term opium use, even at relatively low doses, is associated with an estimated 86% increase in the risk of death [4]. The mortality rate per million among drug users aged 15 to 64 years in Iran was estimated as 69.1, with the majority of deaths related to opiate use [5], compared to 5.4-48.6 per million in Asia and 22 to 55.9 per million worldwide [1]. 

Several risk factors make opiate users vulnerable to hepatitis C virus (HCV), hepatitis B virus (HBV) and human immunodeficiency virus (HIV) and tuberculosis (TB) infection [6]. Risky behaviors include the use of nonsterilized needles by injection drug users (IDU) and unprotected sexual activities, unsafe tattooing, cupping, blood transfusion or dental procedures in both IDU and non-IDU [1,3]. In addition, lack of access to health services, low socioeducational level, history of imprisonment, social exclusion, homelessness, unemployment, alcohol dependency and having other diseases complicate the picture of infection by HCV, HBV and HIV viruses and their related outcomes in many opiate users [7]. 

The global prevalence of HCV infection among IDU in 2010 was 46.7%, meaning that some 7.4 million of the 16 million IDU worldwide are infected with the hepatitis C virus. The HBV infection rate among IDU is about 14.6%, i.e., 2.3 million IDU are infected with this virus, and 18.9% or 3 million of IDU are living with HIV worldwide [1]. Despite the higher prevalence and transmissibility and the equal or higher health and economic costs of HCV compared to HIV infection, especially among IDU, viral hepatitis received far less attention than HIV-related disease [8].

It is estimated that in Shiraz, a city with a population 1.7 million, there are about 32,520 (13249-71063) opiate users between the ages of 15 and 64 years. There are also about 120 legal public and private harm reduction, drug addiction-related and drop-in centers in Shiraz, but none of them screen opiate users for HCV or HBV infection. Not more than 2% of opiate users are referred annually to the only existing behavioral counseling center (BCC) affiliated with Shiraz University of Medical Sciences for free harm reduction services, and this center screens only IDU with HIV infection for HBV and HCV infection. As a result, most opiate users in the city are not screened for HCV and HBV infection. 

We conducted this study to measure the prevalence and determinants of HCV, HBV and HIV infection in all drug users including IDU and non-IDU in order to respond to the need for accurate, detailed data on the size of the opiate user population at particular risk of HCV and HBV infection. The findings are expected to support efforts toward revision of nationwide policies aimed at developing the best, most appropriate protocols that clinics and harm reduction centers can use to provide services to opiate users.

## Patients and Methods

This seroprevalence and questionnaire-based cross-sectional study was conducted from November 2012 to February 2013. All high-risk persons including persons with a history of drug use or high-risk sexual behaviors who were referred to the BCC affiliated with Shiraz University of Medical Sciences in Shiraz (Fars province, southern Iran) were interviewed face to face privately by a trained clinical psychologist. Participation was voluntary and confidentiality was ensured for all participants. The anonymous, coded questionnaire used to structure the interview consisted of an introductory explanation about the aims of the study, introduction of the researchers, a consent form, and questions regarding demographic data, history of drug use, pattern of sexual activity and other risk factors such as imprisonment, tattooing and cupping. The content and face validity of the piloted questionnaire were evaluated by expert opinion, and its reliability was calculated as 0.71 according to the formula for Cronbach’s alpha by SPSS. 

### Ethics Statement

Written consent forms were gathered of all participants in this study. None of the participants asked us to be given incentives for participation in this study. The content of consent forms was approved by Ethics Committee of the Health Policy Research Center affiliated with Shiraz University of Medical Sciences and included a brief explanation about aims of this study and identifications of researchers and interviewers. The signed consent forms were kept in archive of records of this survey. According to our protocol, all potential participants who declined to participate or otherwise did not participate were eligible for treatment (if applicable) and were not disadvantaged in any other way by not participating in the study.

A 10-mL blood sample was obtained from each subject in the BCC laboratory. Blood samples were transferred promptly to the Central Blood Transfusion Organization in Shiraz with appropriate safeguards to maintain cold chain standards. Samples were tested for hepatitis B surface antigen (HBS Ag), antibody to hepatitis B core antigen (HBc Ab), antibody to hepatitis C virus (HCV Ab) and HIV. Hepatitis B e-antigen (HBe Ag) and antibody to hepatitis B e-antigen (HBe Ab) were also tested in persons who were positive for HBS Ag or HBc Ab. A third-generation enzyme linked immunosorbent assay (ELISA) was used to measure HBS Ag and HCV Ab (DIA.PRO kit, Italy). Positivity for HBc monoclonal antibody was determined by ELISA (Siemens Enzygnost, Germany). Western blot assays (MP Diagnostic HCV Blot 3.0 kit and MP Biomedicals HIV kit, Germany) were used as confirmatory diagnostic assays for HCV and HIV infection, respectively. 

All data were entered into SPSS version 11.5 software (SPSS, Chicago, IL, USA). The accuracy of data entry was ensured by randomly selecting and checking completed questionnaires against their corresponding data in the SPSS software. Correlations between interval and nominal variables were determined as the Eta coefficient. Chi-square, Fisher’s exact and *t* tests were used as appropriate. After univariate analysis, cumulative correlations of the variables with *P* ≤ 0.2 (as independent variables) and HBS, HCV or HIV positivity (as dependent variables) were calculated separately for each virus by binary logistic regression (forward model). *P* values less than 0.05 were considered significant. 

## Results

A total of 569 participants (484 men, 85.06%) took part in this study. Their mean age was 30.04 ± 7.79 years; 315 (55.36%) were single, and 426 (74.86%) were high-school educated. One hundred forty-eight (26.01%) were unemployed and 544 (95.6%) lived in the city (Table 1). Injection drug users and non-IDU differed in all demographic characteristics except place of residence (Table 1). Among all participants, 146 (25.65%) were referred to the BCC because of risky sexual behaviors only, compared to 56 (9.84%) who were opiate users only and 323 (56.76%) who were both opiate users and had high-risk sexual behaviors. Two hundred thirty-three (40.94%) used opiates only via injection or both injection and other routes, whereas 145 (25.48%) used these drugs only via non-injection routes.

**Table 1:** Demographic characteristics and risky behaviors in participants referred to the behavioral counseling center affiliated with Shiraz University of Medical Sciences, southern Iran.

**Demographic item**	**All participants**	**IDU†**	**Not IDU**	**P value**	**Odds ratio (95% CI)**
	**n=569**	**n=233**	**n=336**		
**Age (years)**					
Mean	30.04 ± 7.79	32.30±7.33	28.49± 7.72	< 0. 001	CI t (-5.08- -2.54)
Median	29	30	27		
Minimum	13	19	13		
Maximum	59	59	56		
**Gender**					
Male	484 (85.2%)	229 (98.3%)	255 (75.9%)	-	17.85(6.49-50)
Female	84 (14.8%)	4 (1.7%)	80 (23.8%)		
**Level of education**					
Primary school	71 (12.5%)	33 (14.2%)	38 (11.3%)	0.003	-
High school	426 (74.9%)	184 (79%)	242 (72%)		
University	71 (12.7%)	16 (6.9%)	55 (16.4%)		
**Marital status**					
Single	315 (55.4%)	129 (55.4%)	185 (55%)	0.007	-
Married	190 (33.4%)	68 (29.2%)	122 (36.3%)		
Divorced	56 (9.8%)	34 (14.6%)	22 (6.5%)		
Widowed	8 (1.4%)	2 (1%)	6 (1.8%)		
**Place of residence**					
Urban	544 (95.6%)	224 (96.1%)	320 (95.2%)	0.72	0.85(0.36-1.99)
Rural	24 (4.2%)	9 (3.9%)	15 (4.5%)		
**Job status**					
Student	27 (4.7%)	1 (0.4%)	26 (7.7%)	< 0. 001	-
Unemployed	148 (26%)	62 (26.6%)	86 (25.6%)		
Unspecialized worker	32 (5.6%)	21 (9%)	11 (3.3 %)		
Employed	23 (4%)	6 (2.6%)	17 (5%)		
Free Job (Excluding unspecialized workers)	338 (59.6%)	143 (61.4%)	195 (58%)		
**History of imprisonment (years)**					
Yes	248 (43.6%)	181 (77.7%)	67 (19.9%)	< 0. 001	13.88(9.25-20.83)
No	320 (56.4%)	52 (22.3%)	268 (79.8%)		
**Sexual behavior**					
Heterosexual	369 (65%)	144 (61.8%)	225 (67%)	0.21	-
Bisexual	84 (14.8%)	41 (17.6 %)	43 (12.8%)		
Homosexual	15 (2.6%)	4 (1.72%)	11 (3.3%)		
**Condom use in sexual contacts**					
Yes, always	69 (12.3%)	26 (11.2%)	43 (12.8%)	0.59	-
Yes, occasionally	235 (41.3%)	93 (39.9%)	142 (42.3%)		
No	172 (30.2%)	72 (30.9%)	100 (29.8%)		
**Cigarette smoking**					
Yes	434 (76.3%)	223 (95.7%)	211 (62.8 %)	< 0. 001	13.15(6.71-25.64)
No	134 (23.7%)	10 (4.3%)	124 (36.9 %)		
**Tattooing**					
Yes	216 (38%)	120 (51.5%)	96 (28.6%)	< 0. 001	2.68 (1.89-3.81)
No	350 (61.7%)	111(47.6 %)	239(71.1 %)		
**Cupping**					
Yes	104 (18.3%)	52 (22.3%)	52 (15.5%)	0.037	1.57(1.02-2.4)
No	463 (81.5%)	180 (77.3%)	283 (84.2 %)		

† **IDU: Intravenous drug Users**

Needle sharing for drug injection was reported by 91 participants (15.99%). Two hundred forty-eight (43.58%) had history of imprisonment, with a significant difference between IDU and non-IDU (*P*< 0.05). Three hundred sixty-nine (64.85%) were heterosexual, 84 (14.76%) were bisexual and 15 (2.63%) were homosexual. Only 69 (12.12%) used a condom regularly in their sexual contacts. The prevalence of high-risk sexual activity did not differ significantly between IDU and non-IDU (*P* = 0.21). Two hundred sixteen participants (37.96%) had history of tattooing and 104 (18.27%) had history of cupping (a treatment in which evacuated glass cups are applied to intact or scarified skin in order to draw blood toward or through the surface). Among IDU compared to non-IDU, tattooing (51.5% vs 28.57%) and cupping (22.31% vs 15.47%) were more common (P< 0.05). Two hundred twenty-one of all participants (38.84%) were blood donors and 60 (10.54%) had received blood transfusions for different reasons. For hundred twenty-one (73.98%) had a history of dental procedures. Two hundred fifteen (37.78%) mentioned that they would continue to practice high-risk behaviors. 

Ninety-two of the participants (16.16%) and 26 (11.15%) of IDU were vaccinated for HBV. Fifty-five (9.66%) had been screened before for HBV or HCV. Thirty-six (6.32%) stated that their first-degree relatives were vaccinated against HBV. Two hundred three (35.67%) were unaware that there is a vaccine against HBV, and 74 (13%) did not know where they could obtain vaccination. 

Twenty-five participants (4.39%) had previously attended harm reduction centers. Thirteen (2.28%) had received previous treatment for HBV or HCV infection. Eighteen (3.16%, 95% CI: 1.72% - 4.6%) were HBS Ag-positive and 72 (12.65%, 95% CI: 9.92% - 15.38%) were HBc Ab-positive. The HCV antibody was detected in 109 (19.15%, 95% CI: 15.92% - 22.38%) and HIV infection was detected in 23 (4%, 95% CI: 2.39% - 5.61%) (Table 2). In IDU, positivity rates were higher than in non-IDU for HBS Ag (5.57% vs 1.48%), HBc Ab (22.74% vs 5.65%), HCV-Ab (40.34% vs 4.46%) and HIV (7.72% vs 1.48%) (*P* < 0.05) (Table 2). Co-infections were detected at the following rates: HCV/HBV (7.2%, 95% CI: 5.08% - 9.32%), HCV/HIV (3.16%, 95% CI: 1.72% - 4.6%), HBV/HIV (2.63%, 95% CI: 1.32% - 3.94%) and HCV/HBV/HIV (2.46%, 95% CI: 1.32% - 3.94%) (Table 2). 

**Table 2:** Hepatitis and HIV markers in participants referred to the behavioral counseling center affiliated with Shiraz University of Medical Sciences, southern Iran.

**Viral marker**	**All participants**	**IDU†**	**Not IDU**	**P value**	**Odds ratio (95% CI)**
	**n=569**	**n=233**	**n=336**		
**HBS Ag**					
Yes	18 (3.2%)	13 (5.6%)	5 (1.5%)	0.006	3.9(1.37-11.09)
No	550 (96.7%)	220 (94.4%)	330 (98.2%)		
**HBc Ab**					
Yes	72 (12.7%)	53 (22.7%)	19 (5.6%)	< 0. 001	4.89(2.81-8.53)
No	496 (87.2%)	180 (77.2%)	316 (94%)		
**HBe Ag**					
Yes	3 (0.5%)	2 (0.8%)	1 (0.3%)	-	2.89 (0.26-32.07)
No	565 (99.3%)	231 (99.2%)	334 (99.4%)		
**HBe Ab**					
Yes	16 (2.8%)	11 (4.7%)	5 (1.5%)	0.022	3.27(1.12-9.54)
No	552 (97%)	222 (95.3%)	330 (98.2%)		
**HCV Ab**					
Yes	109 (19.2%)	94 (40.3%)	15 (4.5%)	< 0. 001	14.42(8.07-25.77)
No	459 (80.7%)	139 (59.7%)	320 (95.2%)		
**HIV**					
Yes	23 (4%)	18 (7.7%)	5 (1.5%)	< 0. 001	5.52(2.02-15.1)
No	545 (95.8%)	215 (92.3%)	330 (98.2%)		
**HBS Ag / Hbc**					
Yes	12 (2.1%)	8 (3.4%)	4 (1.2%)	-	2.94(0.87-9.88)
No	557 (97.9%)	225 (96.6%)	331 (98.5%)	-	
**HCV/HBV**					
Yes	41 (7.2%)	35 (15%)	6 (1.8%)	< 0. 001	9.69 (4-23.45)
No	527 (92.6 %)	198 (85%)	329 (97.9%)		
**HCV/ HIV**					
Yes	18 (3.2%)	15 (6.4%)	3 (0.9%)	-	7.61 (2.17-26.61)
No	550 (97.4%)	218 (93.6%)	332 (98.8 %)	-	
**HBV/HIV**					
Yes	15 (2.63%)	11 (4.7%)	4 (1.2%)	-	4.1 (1.28-13.3)
No	554 (97.4%)	222 (95.3%)	331 (98.5%)	-	
**HBV/HCV/HIV**					
Yes	14 (2.5%)	11 (4.7%)	3 (0.9%)	-	5.48 (1.51-19.87)
No	554 (97.4%)	222 (95.3%)	332 (98.8%)		

Univariate analysis showed that HCV positivity was more frequent in males (Odds ratio [OR] = 21.27, CI 95% OR = 3.38 - 125), in persons with a history of imprisonment (OR = 5.73, CI 95% OR = 3.81 - 8.63) and patients with tattooing (OR = 9.09, CI 95% OR = 1.04 - 71.42) compared to their counterparts without these antecedents. Markers of HBV infection were more prevalent in males (OR = 3.7, CI 95% OR = 1.28 - 10.1), persons with history of imprisonment (*P* < 0.05, OR = 6.94, CI 95% OR = 3.84 - 12.5) and patients with tattooing (*P* = 0.007, OR = 1.93, CI 95% OR = 1.19 - 3.12) compared to their counterparts. No significant correlation was found between HCV infection and risky sexual behaviors. 

Univariate analysis showed that HIV infection was more common in patients who were illiterate or had a low educational level (*P* = 0.008, OR = 3.33, CI 95% OR = 1.31 - 8.33), patients with history of imprisonment (OR = 9.25, CI 95% OR=2.73 - 31.25), cigarette smokers (OR = 7.14, CI 95% OR = 0.95 - 52.63), patients with tattoos (*P* = 0.022, OR = 2.63, CI 95% OR = 1.12 - 6.25) and in those who had a tendency to continue their high-risk behavior (OR = 3.05, CI 95% OR = 1.02 - 9.10). Binary logistic regression showed that only history of imprisonment was a statistically significant determinant of positivity for HCV Ab in western blot assays (constant = 1.62, ß = -2.19, *P* = <0.05, Exp ß=0.11, CI 95% for Exp ß = 0.06 - 0.18) and positivity for HBV markers (constant = 1.18, ß = -1.92, *P* = <0.05, Exp ß = 0.14, CI 95% for Exp ß = 0.08 -0.26). Binary logistic regression also showed that history of imprisonment (constant = -0.5, ß = -2.29, *P* = <0.05, Exp ß = 0.10, CI 95% for Exp ß = 0.02 - 0.34), history of tattooing (ß = 1.35, P = 0.01, Exp ß = 3.85, CI 95% for Exp ß = 1.26 - 11.75) and low educational level (ß = -1.06, *P* = 0.03, Exp ß = 0.34, CI 95% for Exp ß = 0.13 - 0.90) were statistically significant determinants of HIV positivity. Most participants with evidence of HBV (63 of 78; 80.76%) or HCV infection (91 of 109; 83.48%) belonged to the HIV-negative subgroup. However, except for HBS Ag and HBe Ag, HIV-positive patients had evidence of HBV or HCV infection significantly more frequently than HIV-negative patients (*P* < 0.05).

## Discussion

Our findings speak strongly for the need to screen opiate users, including non-IDU and especially IDU regardless of whether they are positive for HIV infection or not, for HBV and HCV infection. Illicit drug use takes a heavy toll worldwide. According to the World Drug Report by the United Nations Office on Drug and Crime (UNODC), 230 million people, or 1 in 20 adults, are estimated to have used an illicit drug at least once in 2010. This includes 12 to 21 million opiate users or 0.3% to 0.5% of the world population aged 15-64 years, and 15.9 (11.0-21.2) million IDU [3]. Around 60% of all problem drug users worldwide inject drugs, and IDU account for about 7.5% [3] to 8.4% [8] of all drug users worldwide. The number of drug users has been predicted to increase by a quarter before 2050, and developing countries will suffer more than developed countries in this regard [1]. More than half of the world’s opiate users are in Asia [3]. In the Middle East region of Asia 1,890,000 to 3,820,000 people were opiate users in 2008 [7]. According to experts, the opiate use rate in Iran has increased in recent years [3,9] . Iran accounted for 42% (452 tons) of the estimated global opium consumption in 2008, making this country the largest opium consumer worldwide, and the consumption of 17 tons heroin in that same year accounted for 5% of global heroin use [6,10]. The annual prevalence of opiate use (including heroin and opium) in Iran in 2010 was about 2.7% (1.1%-5.9%) of the 53.2 million population between 15 and 64 years of age, and 40% of the estimated opiate users in this country consume opium, while the rest mainly consume heroin [1-3]. In Iran, the rate of drug-related deaths was 69.1 per 1 million people aged 15-64 years [5] compared to 22 to 55.9 deaths per one million inhabitants in this age range worldwide [1]. 

Risky injecting practices by IDU as an extreme form of illicit drug use, and high-risk sexual behaviors in IDU and non-IDU are major public health concerns because of the transmission of blood-borne infections such as HCV, HBV and HIV. This situation has serious health implications and leads to substantial costs for to individuals and the community [3]. 

### HCV

Viral hepatitis clearly poses a challenge to public health. Globally, 3–4 million people are infected with HCV every year; 150 million people are chronically infected, and more than 350,000 people die every year from HCV-related liver disease [11]. In developed countries injection drug use is the main route of HCV transmission. The hepatitis C virus is more infectious than HIV and five times more widespread worldwide [3]. There is a wide range of reported prevalences of HCV infection among IDU in different geographical regions of world. The differences in these estimates reflect uncertainties resulting from variations in prevalence among different subpopulations of IDU [7]. According to the 2012 World Drug Report, the global prevalence of HCV infection among IDU was 46.7% in 2010 [1]. However, this figure was reported as 50.3% in the 2011 World Drug Report [3]. One global systematic review showed that the prevalence of HCV infection among IDU was 60% to 80% in 26 countries and >80% in 12 countries, with a midpoint prevalence of 67% among all IDU globally [8]. The above figures show that there are 7.4 to 16 million IDU worldwide with HCV infection [1]. The largest HCV-positive IDU populations were believed to be living in Eastern Europe (2.3 million; range 1.2–3.9 million) and East and South-East Asia (2.6 million; range 1.8–3.6 million). China, (1.6 million), the USA (1.5 million) and the Russian Federation (1.3 million) had by far the largest such populations [8]. 

In Iran, no large changes were seen between 2008 and 2009 in the reported trend in the prevalence of HCV infection among IDU [3]. A global systematic review showed that the HCV infection rate among IDU in Iran was 50.2% (34.5% to 65.9%) in 2007 [8]. One study in Tehran, Iran reported an HCV infection rate of 34.5% in IDU compared to 40.3% in our findings [12]. In another study, the prevalence of HCV infection among drug users referred to a BCC in Hamadan province (western Iran) was 35.6% between 2005 to 2007 [13]. These prevalence figures are higher than the 19.15% found in the present study. One reason for these differences may be differences between subpopulations in different settings. 

### HBV

Two billion people worldwide have been infected with HBV and about 600,000 people die every year from the acute or chronic consequences of this virus [14]. According to the 2012 World Drug Report, the global prevalence of HBV infection among IDU was 14.6% in 2010 [1]. However, it was reported as 22% in the 2011 World Drug Report [3]. One global systematic review reported that in 2010, HBS Ag infection among IDU ranged from 5% to 10% in 21 countries and was over 10% in 10 countries [8]. Worldwide, 6.4 (2.3–9.7) million IDU might be anti-HBc-positive and 1.2 (0.3–2.7) million are HBs Ag-positive [8]. In low-intermediate prevalence countries, the prevalence of HBs Ag in IDU was typically <10%, whereas in high HBV prevalence countries (e.g. East and South-East Asia), the prevalence of HBs Ag among IDU was on the order of 10% to 20% [8]. The HBs Ag prevalence among IDU in Iran reportedly ranged from 3.7% to 30.9% [8]. One study in Tehran reported that 50.7% of IDU were positive for HBV infection [12], compared to 5.57% in the present study. Another report placed the prevalence of HBV infection among drug users referred to a Hamadan province BCC between 2005 to 2007 at 2.9%, a much lower figure than the 12.65% in the present study [13]. In a survey [15] conducted in 2007 at the Shiraz BCC, the prevalence of HBS seropositivity among patients with HIV infection was 7.45%, which is less than the 8.69% (2 of 23 HIV-positive participants) in the present study. The large differences between studies may be the result of heterogeneity in the subpopulations studied in different geographical areas. 

### HIV

Globally, the prevalence of HIV infection among IDU was estimated as 17.9% in 2009 [3] and 18.9% in 2010 [1]. According to other reports, 3 (0.8–6.2) million IDU (1 of 5 IDU) are living with HIV worldwide [1,7]. There are large geographical variations in the prevalence of HIV among IDU, with the largest numbers and highest rates in Latin America, East Europe, and East and South-East Asia. Combined, these regions account for 73% of the global number of IDU living with HIV. In some countries the prevalence of HIV among IDU is extremely high, such as in Estonia (72%), Argentina (50%) and Brazil (48%) [3]. In Iran, a decrease in the reported prevalence of HIV among IDU was seen in 2009 compared to 2008 [3]. The rate of HIV infection among IDU who participated in the present study was 7.72%, a figure lower than the 10.7% rate in another study in Tehran [12]. In an earlier study at a Hamadan province BCC [14], the prevalence of HIV infection among drug users referred to this center between 2005 and 2007 was 4%, i.e. the same as in the present study in Fars province. 

### HBV/HCV/HIV co-infection

The prevalence of HBV/HCV/HIV co-infection in IDU who participated in our study was 4.72%, compared to 7.1% in China [16], 6.5% in Tehran [12] and 1.3% in male IDU in a city in central Iran [17]. HBV/HCV, HCV/HIV and HBV/HIV co-infection rates in IDU in the present study were 15.02%,6.43% and 4.72% respectively, compared to 21%, 8.7%, and 7.8% in the Tehran study [12]. An earlier study at the Shiraz BCC found that 18.31% of the participants with HCV infection had also HIV infection [18]; this figure is slightly higher than the 16.51% we found in 2012 and 2013. Davarpanah et al. reported in 2004 [15] that the prevalence of HBV/HIV co-infection among persons referred to the Shiraz BCC was 7.5%, about three times the figure we found in the present study. In another study at the Shiraz BCC, the prevalence of HCV/HIV co-infection was 18.31%, more than 2.5 times the rate we detected in this study [18].

### Risk factors for HBV and HCV infection

Different risk factors predispose drug users to HCV and HBV infection. Unsafe injection is one of the most common and important routes of HCV, HBV and HIV infection in IDU, and caused 46.7%, 14.6% and 18.9% respectively of infections by these viruses according to a 2010 report [1]. Unprotected sexual activity, unsafe tattooing and inappropriate cupping procedures are among other risk factors that cause these viruses to be acquired or spread in both IDU and non-IDU in the community. In one study [19] conducted at the Shiraz BCC, risk behaviors among users were injection drug use (40.8% vs 9.84% in the present study), high-risk sexual contact (16.4% vs 25.65%), and both injection drug use and unprotected sexual contact (32.6% vs 56.76%). These findings show that the proportion of high-risk sexual contacts among users of the Shiraz BCC has increased in recent years. Our study shows that around half of IDU had a history of sharing needles, a larger proportion than the 31% found in a study in Isfahan (central Iran) [20]. We also found that more than half of drug users had risky sexual contacts, two third were heterosexual, about 15% were bisexual and around 3% were homosexual, and most of them did not use a condom regularly in their sexual contacts. About half of them had tattoos, i.e. about six-fold as many as the 7.9% found in 2008 by Davarpanah et al. at the Shiraz BCC [19] and three-fold as many as Keramat et al. reported for users of a Hamadan BCC [14]. These figures show an increasing tendency toward tattooing among drug users in recent years. About 11.15% of IDU had been vaccinated for HBV compared to 33.1% of Australian IDU [21]. Therefore increasing HBV vaccine coverage in IDU should be considered. In addition, around three fourths of drug users with HBV or HCV infection (or both) had history of dental procedures before their infection was detected. This finding necessitates better knowledge of infection control and safer practices by dentists in our region. About half of drug users and especially IDU in the present study had a history of imprisonment, as was also found at a Hamadan BCC [14] and in a study in Tehran by Mir-Nasseri et al. [22]. All these lines of evidences show that most drug users, including non-IDU, have multiple risk factors. Among these high-risk groups, less educated drug users who have spent time in prison or have tattoos should be considered a high-priority population for screening and harm reduction for HBV and HCV infection, regardless of their route of drug use and regardless of whether they are HIV-positive or not. 

### Limitations

Our study was not free from potential limitations. We were able to study only a convenience sample of opiate users. Opiate users referred to the Shiraz BCC for harm reduction services may be more concerned about their health compared to others who are not referred to this center. We were aware of this limitation from the beginning of this study. However, this sampling method was unavoidable because many opiate users are beyond the reach of health care and social services. The same limitation applies to many published studies designed with aims similar to ours. Other limitation was that due to very high cost of molecular assays, we could not check HBV-DNA or HCV-RNA in serological positive patients, to detect those who need treatment. 

In conclusion, Iran, a country with a very high rate of opiate consumption, has a large number of opiate users with HBV and HCV infection. No more than half of the estimated number of drug users are receiving care at private and public drug detoxification and harm reduction centers in Shiraz. Meanwhile only 2% of the opiate user population is referred to the BCC. Among all high-risk groups referred to the BCC, around 4% are HIV-positive and only this subgroup is tested for HBV and HCV infection. Therefore, more than 99% of opiate users remained unscreened for these viruses. This situation presents our region with a poor outlook and looming crisis regarding epidemics of HBV and HCV infection. Evidence from this study shows that the current policy of screening only HIV-positive drug users for HBV and HCV infection at the BCC in each province in Iran does not identify most of cases of these infections. Although maintaining and strengthening the response to HIV among IDU remains crucial, the significance of viral hepatitis should receive greater recognition than is currently the case. Therefore, urgent revision of the nationwide protocol should be considered by the Ministry of Health of Iran, and all opiate users should be screened for HBV and HCV infection. This need is concordant with the declaration produced by the WHO 63rd World Health Assembly in May 2010 that countries should incorporate “policies, strategies and tools recommended by WHO in order to define and implement preventive actions, diagnostic measures and the provision of assistance to the population affected by viral hepatitis” including vulnerable populations [8,23]. 
